# Japanese encephalitis virus infection in non-encephalitic acute febrile illness patients

**DOI:** 10.1371/journal.pntd.0008454

**Published:** 2020-07-14

**Authors:** Chairin Nisa Ma’roef, Rama Dhenni, Dewi Megawati, Araniy Fadhilah, Anton Lucanus, I Made Artika, Sri Masyeni, Asri Lestarini, Kartika Sari, Ketut Suryana, Frilasita A. Yudhaputri, Ungke Anton Jaya, R. Tedjo Sasmono, Jeremy P. Ledermann, Ann M. Powers, Khin Saw Aye Myint

**Affiliations:** 1 Emerging Virus Research Unit, Eijkman Institute for Molecular Biology, Jakarta, Indonesia; 2 Faculty of Medicine and Health Sciences, Warmadewa University, Denpasar, Bali, Indonesia; 3 School of Anatomy, Physiology and Human Biology, University of Western Australia, Perth, Australia; 4 Department of Biochemistry, Faculty of Mathematics and Natural Sciences, Bogor Agricultural University, Bogor, Indonesia; 5 Wangaya General Hospital, Denpasar, Bali, Indonesia; 6 Eijkman-Oxford Clinical Research Unit, Eijkman Institute for Molecular Biology Jakarta, Indonesia; 7 Dengue Research Unit, Eijkman Institute for Molecular Biology Jakarta, Indonesia; 8 Division of Vector-Borne Diseases, Centers for Disease Control and Prevention, Fort Collins, Colorado, United States of America; University of Rhode Island, UNITED STATES

## Abstract

Although Japanese encephalitis virus (JEV) is considered endemic in Indonesia, there are only limited reports of JEV infection from a small number of geographic areas within the country with the majority of these being neuroinvasive disease cases. Here, we report cases of JEV infection in non-encephalitic acute febrile illness patients from Bali, Indonesia. Paired admission (S1) and discharge (S2) serum specimens from 144 acute febrile illness patients (without evidence of acute dengue virus infection) were retrospectively tested for anti-JEV IgM antibody and confirmed by plaque reduction neutralization test (PRNT) for JEV infection. Twenty-six (18.1%) patients were anti-JEV IgM-positive or equivocal in their S2 specimens, of which 5 (3.5%) and 8 (5.6%) patients met the criteria for confirmed and probable JEV infection, respectively, based on PRNT results. Notably, these non-encephalitic JE cases were less likely to have thrombocytopenia, leukopenia, and lower hematocrit compared with confirmed dengue cases of the same cohort. These findings highlight the need to consider JEV in the diagnostic algorithm for acute febrile illnesses in endemic areas and suggest that JEV as a cause of non-encephalitic disease has likely been underestimated in Indonesia.

## Author summary

Japanese encephalitis virus (JEV) is an important cause of central nervous system (CNS) infections in Asia and is considered endemic in Indonesia. However, reports of JEV infection in non-encephalitic disease cases are lacking because diagnosis is difficult to confirm and JEV is rarely considered as a cause of non-encephalitic disease. Here, with robust serological testing, we identified cases of JEV infection in patients presenting at a regency hospital in Bali with fever but without symptoms of CNS infection. This finding supports the need to include JEV in routine clinical diagnostic algorithms for patients with fever in endemic areas.

## Introduction

Japanese encephalitis (JE) is a vector-borne disease caused by JE virus (JEV), a single-stranded RNA flavivirus that is transmitted through a zoonotic cycle between mosquitoes, pigs and water birds, with humans as dead-end hosts. JEV is the primary vaccine-preventable cause of encephalitis and the major cause of viral encephalitis in Asia [1]. Clinical outcomes of symptomatic JEV infection may vary from a mild non-specific febrile illness to a severe form of neuroinvasive disease carrying a high mortality rate (20–30%). However, most human infections with JEV are asymptomatic, with only 1% of JEV-infected patients proceeding to develop symptomatic clinical disease [1].

JEV is considered endemic throughout Indonesia, as suggested by early serological studies in animals and humans [2–9], as well as from hospital-based surveillance for acute encephalitis syndrome (AES) [10,11]. Cases of JEV infection in international tourists who have traveled to Bali, Indonesia have also been reported [12–15]. The presence of the virus in Indonesia has been confirmed by virus isolation from local mosquitos and pigs [16–20]. However, isolates from confirmed human cases have yet to be reported.

Studies from Indonesia have reported that death occurred in 10−16% of laboratory-confirmed JE cases while 31−37% of the survivors had neurological sequelae at hospital discharge [10,11]. Long-term assessment of Indonesian children with JE disease showed that half of the children were either dead or left with serious disability [21]. While the AES cases are well described, there is a limited description of symptomatic JEV infections without encephalitis. A study from Thailand reported that 14% of adults with acute undifferentiated fevers but without neurologic deficits were serologically diagnosed as JEV-infection [22]. It is likely that JEV is also an under-recognized cause of fever in Indonesia. Here, with robust serological testing we report cases of JEV infection in non-encephalitic acute febrile illness patients from Bali, Indonesia.

## Materials and methods

### Ethics statement

This study was approved by the Medical Research Ethics Committee of Faculty of Medicine, Udayana University, Bali (ethical approval no. 98/UN.14.2/Litbang/2015 and 1452/UN.14.2/Litbang/2015) and the Eijkman Institute Research Ethics Commission (ethical approval no. 66). Written informed consent for participating in the study was obtained from all patients or the parents/guardians.

### Study site, patient recruitment, and sample collection

Archived patient samples from a cross-sectional prospective study of dengue and acute febrile illness conducted in Wangaya General Hospital (Rumah Sakit Umum Daerah Wangaya) were analyzed. Wangaya General Hospital is located in Denpasar municipality of Bali province. This facility includes emergency room and intensive care units as well as inpatient wards and outpatient polyclinics for a wide variety of diseases. The hospital has 200 beds, 170,000 annual visits, and serves a population of 880,000. Inpatient admissions come from both emergency room and outpatient polyclinics. The leading diagnosis of patients admitted to emergency room, inpatient, and outpatient care were unspecified fever, dengue hemorrhagic fever, and diabetes mellitus, respectively. The study was conducted from March to May 2015 and September 2015 to June 2016 to study dengue and other acute febrile illnesses with the details of enrollment criteria and dengue data already reported [23]. In brief, patients presenting at the hospital with acute febrile illness (fever ≥38°C with onset ≤7 days) but without history of chronic illnesses, human immunodeficiency syndrome, cardiac disease, sepsis, local infections (e.g. cellulitis, abscess), or gastrointestinal and respiratory symptoms were enrolled after informed consent was signed. Patients were recruited by the clinical staff by routine clinical assessment, history taking, physical examination, and laboratory tests on enrollment and/or after initial investigation. Admission blood samples (S1) were collected during patient admission while discharge blood samples (S2) were collected whenever patients were discharged from the hospital. Demographic data of the patients and clinical information were collected at the initial admission and before discharge from the hospital.

### DENV NS1 antigen detection and RT-PCR assays

DENV infection was excluded by testing of the S1 sample for DENV NS1 antigen via the SD Bioline NS1 rapid test (Alere, Australia) and DENV RNA using the Simplexa Dengue Real-time RT-PCR Kit (Diasorin, Italy) or pan-flavivirus RT-PCR as described previously [23,24]. Patients who were confirmed to have acute DENV infection were excluded from the study. Furthermore, pan-alphavirus RT-PCR was also performed to exclude chikungunya virus (CHIKV) infection as previously described [24].

### Anti-JEV and anti-DENV IgM ELISA

The presence of anti-JEV IgM in S2 specimens was detected using JEV IgM antibody capture ELISA (JEV MAC-ELISA), developed by the U.S. Centers for Disease Control and Prevention (CDC) as previously described [25]. Ratios of test serum sample optical density to negative control values (P/N) at 450 nm were calculated. Any sample with a P/N >3 was considered positive while P/N values between 2–3 were considered equivocal. Positive and equivocal S2 specimens were further re-tested paired with corresponding S1 specimens to look for seroconversion. The presence of anti-DENV IgM was also tested in both S1 and S2 specimens by using the DENV MAC-ELISA, developed by the Armed Forces Research Institute of Medical Sciences (AFRIMS), Thailand. Serum samples with DENV MAC ELISA binding index results ≥40 U were considered positive [26].

### Plaque reduction neutralization test (PRNT)

All specimens positive or equivocal by JEV MAC-ELISA were tested for the presence of neutralizing antibodies against JEV and DENV by PRNT. PRNT was performed with JEV strain Nakayama, DENV-1 strain PUO-359, DENV-2 strain PUO-218, DENV-3 strain PaH881/88, and DENV-4 strain 1228 as previously described [27]. Briefly, 2-fold serial dilutions of serum were mixed with each challenge virus and incubated at 37°C for 1 hour. The antibody-virus mixture was then inoculated onto baby hamster kidney (BHK-21) cell monolayers for 5 days before plaques were counted. The neutralization titer was expressed as the inverse of the maximum serum dilution yielding a ≥90% reduction in plaque number (PRNT_90_).

The diagnosis was classified as either confirmed JEV, probable JEV, DENV, or flavivirus infection (Table 1, adapted from [28]). Confirmed JEV infection was defined as those who were anti-JEV IgM-positive/equivocal in the S2 and/or S1 specimen with ≥4-fold increased anti-JEV PRNT_90_ titer in S2 from S1 and an anti-JEV PRNT_90_ titer ≥4-fold higher than any anti-DENV titer in S2. Probable JEV infection was defined as those who were anti-JEV IgM-positive/equivocal in the S2 and/or S1 specimen with increased anti-JEV PRNT_90_ titer in S2 from S1 specimen but did not have an anti-JEV PRNT_90_ titer ≥4-fold higher than any anti-DENV titer in S2 specimen and were anti-DENV IgM-negative in both S1 and S2 specimen. DENV infection was defined as patients who were anti-JEV IgM-positive/equivocal in the S2 and/or S1 specimen but with increased anti-DENV PRNT_90_ titer in S2 from S1 specimen alongside an anti-DENV PRNT_90_ titer ≥4-fold higher than anti-JEV titer in S2 specimen and anti-DENV IgM-positive in the S1 and/or S2 specimen. Finally, flavivirus infection was defined as those who were anti-JEV IgM-positive/equivocal in the S2 and/or S1 specimen but anti-JEV PRNT_90_ titer in S2 <4-fold than any anti-DENV titer and with anti-DENV IgM-positive in the S1 and/or S2 specimen. The designation “probable JEV” was used because of the extensive cross-reactivity in secondary flavivirus infections; i.e. neutralizing antibody titers may be higher against a previous flavivirus infection rather than the most recent heterologous flavivirus infection as shown in other studies [29–33].

**Table 1:** Diagnostic interpretation of the serology testing results.

Diagnosis	Criteria
Confirmed JEV	Anti-JEV IgM-positive/equivocal in the S2 and/or S1, and
	Anti-JEV PRNT_90_ titer in S2 ≥4-fold higher from S1, and
	Anti-JEV PRNT_90_ titer in S2 ≥4-fold higher than any DENV titer
Probable JEV	Anti-JEV IgM-positive/equivocal in the S2 and/or S1, and
	Anti-JEV PRNT_90_ titer in S2 higher from S1, but
	Anti-JEV PRNT_90_ titer in S2 <4-fold than any anti-DENV titer, and
	Anti-DENV IgM-negative in the S1 and S2
DENV	Anti-JEV IgM-positive/equivocal in the S2 and/or S1, but
	Anti-DENV PRNT_90_ titer in S2 higher from S1, and
	Anti-DENV PRNT_90_ titer in S2 ≥4-fold higher than JEV titer, and
	Anti-DENV IgM-positive in the S1 and/or S2
Flavivirus	Anti-JEV IgM-positive/equivocal in the S2 and/or S1, but
	Anti-JEV PRNT_90_ titer in S2 <4-fold than any anti-DENV titer, and
	Anti-DENV IgM-positive in the S1 and/or S2

S1, admission serum sample; S2, discharge serum sample; PRNT, plaque reduction neutralization test.

### Virus isolation

Virus isolation was attempted by inoculating patient S1 serum onto African green monkey kidney Vero cells as previously described [24]. Cells were observed daily for cytopathic effects for up to 10 days.

### Statistical analysis

Statistical analysis was performed using OpenEpi v3.01 and GraphPad Prism v8. Quantitative data differences between groups were compared by unpaired Student t test for normally distributed data (based on D’Agostino-Pearson normality test) or by Mann-Whitney test for non-Gaussian distributed data. Categorical data were compared using Mantel-Haenszel chi-square test when all expected numbers are at least 1 or otherwise by using Fisher’s exact test. *P*-values less than 0.05 were considered statistically significant.

## Results

**Fig 1:**
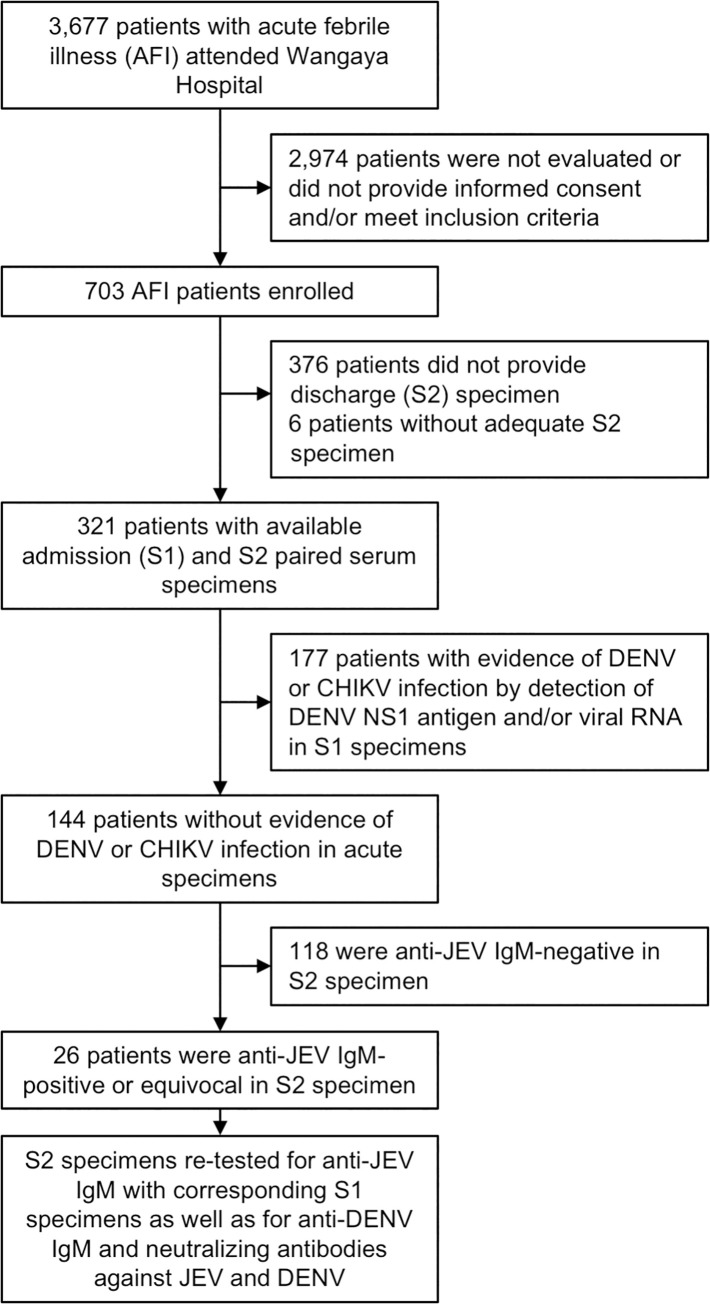
Patient enrollment, specimens collected, and molecular and serological test performed. DENV NS1 antigen was detected by using SD Bioline NS1 rapid test while DENV RNA was detected using Simplexa Dengue Real-time RT-PCR Kit or pan-flavivirus RT-PCR. CHIKV RNA was detected using pan-alphavirus RT-PCR. Anti-JEV and anti-DENV IgM was detected by ELISA, while neutralizing antibodies against JEV and DENV were detected by plaque reduction neutralization test (PRNT).

During the study period, 3,677 patients with suspected dengue or acute febrile illness attended Wangaya Hospital and a total of 703 patients were enrolled in the study (Fig 1). Of these, 321 patients had both admission (S1) and discharge (S2) paired serum specimens available and were included in this study analysis. One hundred forty-four patients showed no evidence of acute DENV infection (by detection of DENV NS1 Ag and/or RNA) nor CHIKV infection; these were tested for the presence of anti-JEV IgM. Of 144 patients, 26 (18.1%) were anti-JEV IgM-positive or equivocal in their S2 specimens (Fig 1). All 26 were further tested for the presence of anti-DENV IgM and DENV/JEV neutralizing antibodies by PRNT in paired specimens, from which 5 (3.5%) and 8 (5.6%) met the criteria for confirmed JEV and probable JEV infection, respectively. The other 5 (3.5%) and 8 (5.6%) patients were classified as having DENV and flavivirus infection, respectively (Table 2). Cell culture and pan-flavivirus RT-PCR attempted from the S1 specimens did not produce any JEV positive results.

**Table 2:** Serology testing results from non-encephalitic acute febrile illness patients with confirmed JEV, probable JEV, DENV, and flavivirus infection.

No.	Patient sample ID	Age (years)	Days after onset	IgM		PRNT_90_ titer				
				JEV^a^	DENV^b^	JEV	DENV-1	DENV-2	DENV-3	DENV-4
JEV (confirmed)										
1	WGY599 S1	20	3	−	−	40	<10	<10	<10	<10
	WGY599 S2		6	+	+	1,280	20	80	20	20
2	WGY623 S1	32	3	−	−	<10	<10	<10	<10	<10
	WGY623 S2		7	+	+	640	40	40	80	80
3	WGY656 S1	23	2	EQU	−	20	<10	80	10	20
	WGY656 S2		3	+	−	>320	40	40	40	NA
4	WGY726 S1	32	7	+	+	80	10	40	80	10
	WGY726 S2		10	+	+	320	10	40	20	20
5	WGY733 S1	15	4	−	−	80	<10	40	80	20
	WGY733 S2		7	+	+	320	10	40	80	20
JEV (probable)										
6	WGY067 S1	22	4	QNS	−	40	160	80	80	80
	WGY067 S2		7	+	−	80	>2,560	160	160	160
7	WGY143 S1	4	7	QNS	−	40	640	80	80	20
	WGY143 S2		12	EQU	−	160	>2,560	160	160	80
8	WGY317 S1	47	5	+	−	40	<10	80	10	40
	WGY317 S2		8	+	−	80	<10	160	10	80
9	WGY629 S1	19	5	+	−	160	20	160	80	160
	WGY629 S2		7	+	−	320	320	1,280	160	320
10	WGY647 S1	27	5	+	−	80	160	80	40	80
	WGY647 S2		6	+	−	160	1,280	20	160	80
11	WGY658 S1	30	3	EQU	−	<10	40	<10	<10	10
	WGY658 S2		5	EQU	−	80	80	<10	<10	<10
12	WGY667 S1	6	5	EQU	−	20	<10	20	10	40
	WGY667 S2		7	+	−	160	20	160	160	160
13	WGY713 S1	72	3	EQU	−	10	NA	40	NA	NA
	WGY713 S2		8	EQU	−	80	40	320	40	40
DENV										
14	WGY065 S1	33	5	QNS	−	320	160	160	320	160
	WGY065 S2		8	+	+	320	>2,560	>2,560	>2,560	160
15	WGY071 S1	16	4	QNS	−	20	10	40	160	20
	WGY071 S2		7	+	+	20	80	160	>2,560	80
16	WGY321 S1	24	5	−	−	160	160	40	80	20
	WGY321 S2		7	+	+	160	1280	160	160	80
17	WGY724 S1	28	4	EQU	−	<10	40	10	20	10
	WGY724 S2		7	EQU	+	20	80	160	80	80
18	WGY727 S1	19	5	−	+	20	40	40	40	40
	WGY727 S2		6	EQU	+	160	>2,560	80	80	160
Flavivirus										
19	WGY089 S1	33	5	QNS	+	40	20	80	80	40
	WGY089 S2		8	+	+	80	20	80	160	40
20	WGY174 S1	3	4	+	+	40	10	80	80	160
	WGY174 S2		5	+	+	320	10	320	80	320
21	WGY178 S1	20	3	+	−	80	40	80	<10	<10
	WGY178 S2		5	+	+	160	40	320	20	20
22	WGY516 S1	15	3	QNS	+	<10	<10	<10	40	<10
	WGY516 S2		5	EQU	+	20	<10	<10	40	<10
23	WGY575 S1	26	4	EQU	−	20	20	40	20	10
	WGY575 S2		5	+	+	160	40	80	160	40
24	WGY674 S1	21	5	−	−	40	<10	<10	<10	<10
	WGY674 S2		7	+	+	40	10	20	<10	20
25	WGY684 S1	16	5	+	+	80	<10	10	40	10
	WGY684 S2		6	+	+	80	10	20	40	20
26	WGY708 S1	31	3	+	−	NA	NA	NA	NA	NA
	WGY708 S2		6	+	+	40	40	40	20	40

^a^Ratios of samples optical densities divided by negative control were calculated; <2, negative (−); 2–3 equivocal (EQU); >3 positive (+).

^b^Calculated ELISA binding index results ≥40 U were considered positive (+), while <40 U were considered negative (−).

S1, admission serum sample; S2, discharge serum sample; QNS, quantity not sufficient.

Two patients (WGY599 and 623) with confirmed JEV infection diagnosis had no detectable or low neutralizing antibodies to JEV or any DENV in their S1 specimen, suggesting that JEV was the first flavivirus that they encountered (primary flavivirus/JEV). These two patients had IgM-positive results to both JEV and DENV, however a ≥4-fold rise of anti-JEV PRNT_90_ titer and a ≥4-fold difference between anti-JEV PRNT_90_ titer and anti-DENVs titers were observed in their S2 specimen, indicating a low level of cross-reactivity in the cases of primary JEV/flavivirus infection.

Most of the patients with probable JEV diagnosis had neutralizing antibody response to DENV in their S1 sample, suggesting previous exposure to DENV. Although most of these probable JEV-infected patients had a higher neutralizing antibody titer to DENV compared to JEV in their S2 specimen, we believe that these patients were infected with JEV since all had no detectable DENV IgM in both S1 and S2 specimens with positive or equivocal JEV IgM in the S2 specimen. The phenomenon of higher serologic reactivity to the previous flavivirus infection than to the current infection is known as “original antigenic sin” and has been documented in a number of studies in human and animal models [29–33]. The mechanism of this phenomenon is not completely understood but is likely attributed to a preferential expansion of memory B cell clones generated from previous flavivirus infection that cross-react and recognize the current infecting heterologous flavivirus [34]. In addition, the absence of DENV RNA detectable in S1 specimens by highly sensitive RT-PCR provides further evidence of JE diagnosis as opposed to dengue.

Four out of five (80%) patients diagnosed as DENV infection had high PRNT titer (≥1,280) to one or more DENV serotypes with detectable anti-DENV IgM in the S2 specimen. Similar to probable JEV-infected patients, neutralizing antibody against DENV was detectable in their S1 which suggest secondary DENV infection. This was also observed in patients with flavivirus infection diagnosis. However, in the flavivirus group, serological data is not sufficient to distinguish the likely infecting flavivirus.

The clinical characteristics of JEV-infected patients are provided in Table 3 along with 177 confirmed DENV-infected patients identified by DENV NS1 antigen detection and/or RT-PCR from the same study cohort (Fig 1). The age of the JE patients ranged from 4 to 72 years old (median 23 years old) and eight of 13 patients were male (62%) (Table 3). The most notable symptoms were malaise, nausea, and loss of appetite, which was observed in 85%, 69%, and 54% of the patients, respectively. Hematological investigations showed thrombocytopenia and leukopenia in nine (69%) and seven (54%) patients, respectively. Mean nadir thrombocyte count was 107 ± 74.2 × 10^3^/μl (median 94 × 10^3^/μl) while mean nadir leukocyte count was 4 ± 1 × 10^3^/μl (median 4 × 10^3^/μl) among all 13 patients. No neurological manifestations or rashes were reported. Notably, there were no statistically significant differences found in clinical characteristics between confirmed and probable JE cases except for lowest hemoglobin level (14.5 ± 1.3 vs 12.6 ± 1.3 g/dl, *P* = 0.0319).

**Table 3:** Characteristics of patients with JEV and DENV infection identified in this study.

Patient characteristics		JEV (n = 13)^a^	DENV (n = 177)^b^
Demographic			
	Sex, male	8 (62%)	84 (48%)
	Age	23 (4−72)	24 (1−75)
Duration of illness before admission, days		4 (2−7)	4 (2−7)
Hematology			
	Lowest hemoglobin, g/dl	13 (10−16)	13 (9−17)
	Lowest leukocyte counts, 10^3^/μl	4 (2−5)^c^	3 (1−8)
	Leukopenia (<4,000/μl)	7 (54%)^d^	144 (82%)
	Lowest thrombocyte counts, 10^3^/μl	94 (24−258)	53 (0.03−508)
	Thrombocytopenia (<150,000/μl)	9 (69%)^e^	163 (92%)
	Highest hematocrit, %	47 (33−89)^f^	43 (6−164)
Clinical symptoms			
	Malaise	11 (85%)	153 (86%)
	Nausea	9 (69%)	143 (81%)
	Loss of appetite	7 (54%)	125 (71%)
	Myalgia	5 (39%)	87 (49%)
	Headache	4 (31%)^g^	105 (59%)
	Hemorrhage	3 (23%)	28 (16%)
	Arthralgia	2 (15%)	71 (40%)
	Vomiting	2 (15%)	72 (41%)
	Cough	1 (8%)	15 (9%)
	Retro-orbital pain	1 (8%)	38 (22%)
	Rash	0 (0%)	11 (6%)
	Diarrhea	0 (0%)	4 (2%)
	Runny nose	0 (0%)	4 (2%)
	Neck stiffness	0 (0%)	1 (1%)
	Seizure	0 (0%)	0 (0%)
	Abdominal pain	0 (0%)	25 (14%)
	Altered mental status	0 (0%)	0 (0%)
	Paralysis	0 (0%)	0 (0%)
	Glasgow Coma Scale <15	0 (0%)	0 (0%)

Data are presented as number of patients (%) or median (range).

^a^Includes confirmed JEV (n = 5) and probable JEV (n = 8) infection.

^b^Includes confirmed DENV-infected patients identified by detection of DENV NS1 antigen detection and/or RT-PCR as depicted in Fig 1.

^c^Statistically significant compared with DENV (*P* = 0.002).

^d^Statistically significant compared with DENV (*P* = 0.018).

^e^Statistically significant compared with DENV (*P* = 0.007).

^f^Statistically significant compared with DENV (*P* = 0.003).

^g^Statistically significant compared with DENV (*P* = 0.045).

Interestingly when compared with the dengue patients, JE patients were less likely to have leukopenia (54% vs 82%, *P* = 0.018), thrombocytopenia (69% vs 92%, *P* = 0.007), or headache (31% vs 59%, *P* = 0.045). JE patients were also found to have a higher hematocrit than dengue patients (median 47% vs 43%, *P* = 0.030) (Table 3). Furthermore, although our study plans did not include follow up assessment to determine long term outcome of the patients after discharge, all JE patients had resolved symptoms upon hospital discharge.

It is not clear from our data if there was any temporal distribution pattern of the JEV infection since our study was not conducted throughout the whole year and the limited number of identified JE cases prevented making such an analysis. However, out of the 13 JE cases, six cases occurred in May, two cases in July, and each one case in March, April, June, September, and October.

## Discussion

JEV was not previously considered a significant public health problem in Indonesia until nationwide studies in the early 2000s (based on syndromic surveillance and serologic assays) suggested nationwide JEV endemicity [9–11]. Although there are a number of laboratory tests to diagnose JEV infection, virus detection assays are not useful for diagnostic purposes due to low-level, transient viremia, making anti-JEV IgM ELISA the WHO recommended method for JEV diagnosis and surveillance [35]. However, cross-reactive IgM antibodies have been detected in about 10% of DENV and JEV cases [36,37]. Therefore, a conservative case definition was used here to define JEV infection based on IgM ELISA followed by confirmation with PRNT in both admission and discharge serum samples. This study confirmed JEV as a cause of non-encephalitic acute febrile illness in Bali, where both JEV and DENV co-circulate.

In this study population, confirmed and probable JEV infection were identified in 9% (13 out of 144) cases. From the thirteen JE patients diagnosed in this study, eleven were adults while only two were children. Previous studies showed that more than 80% of Indonesian children have experienced DENV infection at least once before the age of ten [38], which likely explains the low prevalence of cases with no detectable or low neutralizing antibodies to JEV or any DENV in their S1 specimen (i.e. primary JEV/flavivirus infection) in our study. Further, the presence of pre-existing DENV antibodies in JEV-infected patients has recently been associated with better patient outcomes [39]. Hence, the absence of severe or encephalitic disease in these subjects could be partly attributed to pre-existing DENV immunity.

Thrombocytopenia, prevalent in DENV-infected patients identified in this study, was also observed in 69% of the febrile JE cases, similar to the non-encephalitic JEV infections from Thailand [22]. Malaise, nausea, loss of appetite, myalgia, and headache were the major symptoms reported in the JEV cases here, similar to those reported previously [22]. However, these symptoms were also present in DENV-infected patients at similar frequency except for headache which was less observed in JE cases. While this study suggests that thrombocytopenia, leukopenia, and lower hematocrit were less likely to be found in non-encephalitic JE compared with dengue cases, further studies are needed to confirm these findings.

JEV is routinely included in the diagnostic algorithm of AES in endemic areas of Indonesia. However, reports of JEV as the cause of non-encephalitic illness by using a virus specific PRNT confirmatory assay are lacking in Indonesia. The use of PRNT in this study was vital in confirming JEV infection especially in cases where anti-JEV and -DENV IgM were both detected as exemplified in patients WGY599, 623, 726, and 733. Unfortunately, there is limited laboratory capacity in Indonesia to perform PRNT or detect flaviviruses other than DENV. The role of other vector-borne viruses, including JEV, as causes of febrile illness or encephalitis has therefore likely been underestimated. As such, JEV remains an important public health concern in Indonesia and the transmission of JEV warrants further investigation.

This study is limited by the number of non-encephalitic JE cases identified which does not allow for a sound stratified analysis of the results particularly regarding the clinical features. Furthermore, the higher prevalence of adults over children identified in the study might be due to lack of appropriate population denominator data. The incidence of non-encephalitic JE might potentially be higher in children if the data from this study were adjusted by age stratified population denominator (i.e. the number of susceptible children during the study period).

In summary, this work demonstrates JEV infection in non-encephalitic acute febrile illness patients identified using robust serological assays. Although JEV vaccination has recently been introduced in Bali [40] with reported coverage of 94% in 2018 [41], it has not been widely implemented throughout Indonesia. Hence, further JEV surveillance is required to fully reveal the epidemiology of JE disease in humans. This report on JEV as the cause of acute febrile illness in Bali is fundamental to characterizing JE epidemiology, identifying high-risk areas, and documenting the impact of prevention measures in Indonesia.
