# Supplementary material for: Japanese encephalitis virus infection in non-encephalitic acute febrile illness patients
Source: PLoS Negl Trop Dis. 2020 Jul 14;14(7):e0008454. doi: 10.1371/journal.pntd.0008454 (PMC7360021; doi:10.1371/journal.pntd.0008454)
Supplement: Attachment — Submitted filename: Comments.docx [file pntd.0008454.s001.docx]

Comments

1. Materials and methods – the location of the study hospital, usual patient flow, type of patients attending the hospital should be described in detail.
2. Materials and methods – did the study hospital provide emergency care, have outpatient and inpatient departments? It is not clear why patients attending emergency and outpatient services were not included. It is mentioned that AFI patients with respiratory or GI symptoms were excluded. However, from the current case definition, it is not clear if AFI patients with infection of other systems (e.g. CNS infection, sepsis) or local infection were excluded. If that is the case, how and when (on admission/after some initial investigation) it was done? How enrolment/recruitment of all patients meeting case definition ensured?
3. Results - I would expect a flowchart showing how many patients with case definition attended the hospital during the study period, what proportion could be enrolled, what proportion provided samples. A large proportion of recruited patients didn’t provide sample, how were these patients different from the patients providing samples? Did the patients with longer stay in hospital (hence severe form of diseases) provide both acute and convalescent samples?
4. Results – how the patients with confirmed and probable JEV infection differed from other patients, specifically patients having dengue in terms of clinical presentation or lab findings? Do the authors consider that the results of this study have some implication for the clinical practitioners?
5. It is important to know about the temporal distribution of the JE patients identified and how compares with other causes of AFI.
6. What was the outcome (at least outcome of hospitalization if a comparatively long term outcome is not available) of these 13 JE confirmed/probable patients?
