# Supplementary material for: Japanese encephalitis virus infection in non-encephalitic acute febrile illness patients
Source: PLoS Negl Trop Dis. 2020 Jul 14;14(7):e0008454. doi: 10.1371/journal.pntd.0008454 (PMC7360021; doi:10.1371/journal.pntd.0008454)
Supplement: Attachment — Submitted filename: Review PLoS NTD April, 2020.docx [file pntd.0008454.s002.docx]

Major findings:

1. LN 111-112 states “Positive and equivocal S2 specimens were further re-tested paired with corresponding S1 specimens to look for seroconversion”. LN 231states that JEV infection was defined by samples that were IgM positive or probable on either S1 or S2 plus PRNT. Among the 28 paired samples analyzed by PRNT, there was no case with S1 IgM+ and S2 IgM-. But, since this was a possibility, why was S1 sample in the original pool of 140 paired sample not tested by IgM MAC ELISA?
2. Please clarify the origin of the differential diagnosis rule where a JEV PRNT titers must be at least 4-fold higher than the DEV titers to be consider JEV seroconversion and not cross-reactivity. Is this a WHO, CDC or like reference?
3. The definition of probable JEV, at least in some cases, seems difficult to defend. Notwithstanding the absence of DEN IgM, the S2 DEN PRNT titers for WGY067, 143 and 647 seem so elevated compared to their corresponding S2 JEV PRNT titers that is difficult not to think of them as clear DEN seroconversions.
4. Limiting virus isolation and RT-PCR to the S1 samples probably limits the manuscript findings.

Minor findings:

1. Why was virus isolation not attempted first in a cell culture system, rather than mosquito inoculation?
2. Please, comment of the state of JEV vaccination in Indonesia in general and the reach of JEV vaccination in Bali.
3. LN 226, The sentence “80% of Indonesian children have experienced DENV infection at least once before the age of ten, which likely explains the low prevalence of primary JEV infection in our study”. Do the authors mean primary DEN?
4. Pg 6. The final assessment of JEV positivity by IgM and PRNT would be better expressed in a table to facilitate reading.
