# Supplementary material for: Japanese encephalitis virus infection in non-encephalitic acute febrile illness patients
Source: PLoS Negl Trop Dis. 2020 Jul 14;14(7):e0008454. doi: 10.1371/journal.pntd.0008454 (PMC7360021; doi:10.1371/journal.pntd.0008454)
Supplement: Attachment — Submitted filename: JEV Manuscript Reviewer Comments Response v3.1.docx [file pntd.0008454.s003.docx]

**Manuscript title:** Japanese Encephalitis Virus Infection in Non-Encephalitic Acute Febrile Illness Patients

Please note that line numbers refer to the clean version of the manuscript.

**Author responses to AE comments:**

1. Please be sure to respond to the reviewer comments sent as separate files.

**Response**: Please see below our responses to the reviewer comments.

2. Please provide a brief description of the collection of clinical and clinical laboratory data (Table 2), e.g., when and how frequently this was collected.

**Response**: Collection of clinical and clinical laboratory data was performed twice (during admission and just before discharge). We have now added this information in lines 118−120.

3. Please comment on whether the clinical characteristics were similar in the cases defined as confirmed and probably JE (Table 2).

**Response**: There were no statistically significant differences found in clinical characteristics between confirmed and probable JE cases except for lowest hemoglobin level (14.5 ± 1.3 vs 12.6 ± 1.3 g/dl, *P* = 0.0319). We have now added this information in lines 274−277.

**Author responses to reviewer comments from “Comments.docx”**

Comments

1. Materials and methods – the location of the study hospital, usual patient flow, type of patients attending the hospital should be described in detail.

**Response**: We thank the reviewer for the suggestion. We have now added this information to the main text lines 100−107.

**Text added**: Wangaya General Hospital (Rumah Sakit Umum Daerah Wangaya) is located in Denpasar municipality of Bali province. This facility includes emergency room and intensive care units as well as inpatient wards and outpatient polyclinics for a wide variety of diseases. The hospital has 200 beds, 170,000 annual visits, and serves a population of 880,000. Inpatient admissions come from both emergency room and outpatient polyclinics. The leading diagnosis of patients admitted to emergency room, inpatient, and outpatient care were unspecified fever, dengue hemorrhagic fever, and unspecified diabetes mellitus, respectively.

2. Materials and methods – did the study hospital provide emergency care, have outpatient and inpatient departments? It is not clear why patients attending emergency and outpatient services were not included. It is mentioned that AFI patients with respiratory or GI symptoms were excluded. However, from the current case definition, it is not clear if AFI patients with infection of other systems (e.g. CNS infection, sepsis) or local infection were excluded. If that is the case, how and when (on admission/after some initial investigation) it was done? How enrolment/recruitment of all patients meeting case definition ensured?

**Response**: The study hospital provides emergency care as well as outpatient and inpatient services as mentioned above. Our JE study described in the manuscript was part of the bigger study investigating dengue and other acute febrile illnesses. Some results of this study have previously been published (Megawati et al., 2017). Although we recruited and collected samples from outpatients as well, we did not include outpatient samples in our JE study since convalescent samples (S2) were not collected from outpatients. Furthermore, we have also added the details of the exclusion criteria. All patients were recruited by the clinical staff according to the selected criteria above by routine clinical assessment, history taking, physical examination, and laboratory tests on enrollment and/or after initial investigation. We have now added this information to lines 107−116.

3. Results - I would expect a flowchart showing how many patients with case definition attended the hospital during the study period, what proportion could be enrolled, what proportion provided samples. A large proportion of recruited patients didn’t provide sample, how were these patients different from the patients providing samples? Did the patients with longer stay in hospital (hence severe form of diseases) provide both acute and convalescent samples?

**Response**: We thank the reviewer for the suggestion. We have now added flowchart figure depicting enrollment, collected samples, and assays performed in this study (Figure 1). All inpatients provided a second sample before discharge regardless of the duration of their stay. As we have mentioned above, although we recruited and collected samples from outpatients as well, we did not include outpatient samples in our JE study since convalescent samples (S2) were not collected from outpatients. Additionally, we would like to correct some of the numbers in the manuscript. Accordingly, from a total of 703 patients enrolled, 321 patients provided admission (S1) and discharge (S2) specimens. Out of 321 patients, 144 had no evidence of acute DENV or CHIKV infection. This information did not change the main conclusion of the study; the main text has now been revised (lines 218−224) and Figure 1 has now been included.

4. Results – how the patients with confirmed and probable JEV infection differed from other patients, specifically patients having dengue in terms of clinical presentation or lab findings? Do the authors consider that the results of this study have some implication for the clinical practitioners?

**Response**: We thank the reviewer for the suggestion. We have now added clinical data from confirmed DENV-infected cases for comparison with JEV cases in Table 3. For statistical purpose, all quantitative data were expressed as median (range) instead of mean (standard deviation) since most dengue data had non-Gaussian distribution. We have also added a discussion on comparison between JEV and DENV-infected patient clinical data (lines 278−284 and 328−336).

5. It is important to know about the temporal distribution of the JE patients identified and how compares with other causes of AFI.

**Response**: We did not include analysis of the temporal distribution of JE as the study was not conducted the whole year. In addition, there were only 13 confirmed/probable JE patients identified to make a conclusive temporal distribution. However, we have now added the information regarding the month of the identified JE cases (lines 285−289).

6. What was the outcome (at least outcome of hospitalization if a comparatively long term outcome is not available) of these 13 JE confirmed/probable patients?

**Response**: Our study plans did not include follow up assessment to determine final outcome of patients after discharge. However, on discharge, symptoms were resolved for all JE patients. We have now added this information in lines 282−284.

**Author responses to reviewer comments from “Review PLoS NTD April, 2020.docx”**

Major findings:

1. LN 111-112 states “Positive and equivocal S2 specimens were further re-tested paired with corresponding S1 specimens to look for seroconversion”. LN 231states that JEV infection was defined by samples that were IgM positive or probable on either S1 or S2 plus PRNT. Among the 28 paired samples analyzed by PRNT, there was no case with S1 IgM+ and S2 IgM-. But, since this was a possibility, why was S1 sample in the original pool of 140 paired sample not tested by IgM MAC ELISA?

**Response**: We did not test the S1 sample in the original pool of 140 paired sample because there is a limited evidence of JE cases with IgM-positive in the acute sample (S1) but negative in the convalescent sample (S2) in the literature. As was shown in other study, anti-JEV IgM can be detected in 88% of patient samples collected on day 9−12 and in 100% of patient samples collected on day 13−21 or later (Chanama et al., 2005). Therefore, testing for anti-JEV IgM in the S1 when the S2 specimens were already negative is not justified.

2. Please clarify the origin of the differential diagnosis rule where a JEV PRNT titers must be at least 4-fold higher than the DEV titers to be consider JEV seroconversion and not cross-reactivity. Is this a WHO, CDC or like reference?

**Response**: This diagnosis definition was based on a study from US CDC Vector-Borne Disease group together with WHO (Robinson et al., 2010). In that study, serum samples with JEV IgM positive or equivocal with JEV PRNT titer ≥4-fold DENV were considered confirmed JEV infection. We have now added this information to indicate that our case definition was adapted based on this reference (line 155).

3. The definition of probable JEV, at least in some cases, seems difficult to defend. Notwithstanding the absence of DEN IgM, the S2 DEN PRNT titers for WGY067, 143 and 647 seem so elevated compared to their corresponding S2 JEV PRNT titers that is difficult not to think of them as clear DEN seroconversions.

**Response**: Detection of serum JEV IgM and JEV PRNT titer ≥10 but without 4-fold difference from DENV PRNT titer is a presumptive JEV infection as suggested by Robinson et al (Robinson et al., 2010). The elevated DENV PRNT titer in probable JEV cases in our study were likely because the patients have previously been infected with DENV. The phenomenon of higher serologic reactivity to the previous flavivirus infection than to the current infection reported as “original antigenic sin” has been widely documented in a number of flavivirus studies in human and animal models (Bradt et al., 2019; Chan et al., 2016; Lanciotti et al., 2008; Makino et al., 1994; Montoya et al., 2018). The mechanism of “original antigenic sin” has not been completely understood but is likely due to preferential expansion of memory B cell clones generated from previous infection that cross react and recognize the current infecting heterologous flavivirus (Wong and Bhattacharya, 2019). We have now added these to the text in lines 243−249.

4. Limiting virus isolation and RT-PCR to the S1 samples probably limits the manuscript findings.

**Response**: Negative virus isolation and RT-PCR in JE cases is not uncommon because of the low level and transient viremia which is cleared soon after onset of illness. Even in cases with neurological diseases, JEV is rarely detected or isolated from the cerebrospinal fluid (Solomon et al., 2000). For these reasons, WHO recommended serological assays for JEV diagnosis and surveillance (World Health Organization, 2006). Additionally, if virus or viral nucleic acid are not detected in the acute specimen (S1), it is highly unlikely that these tests would be positive in a convalescent (S2) sample. We agree that this might be a slight limitation of our study, but this does not negate our findings that JEV is one of the causes of non-encephalitic acute febrile illness in Bali.

Minor findings:

1. Why was virus isolation not attempted first in a cell culture system, rather than mosquito inoculation?

**Response**: We did attempt virus isolation by inoculating patient acute serum onto Vero cells as we have described in lines 179−182 (previously 151−154).

2. Please, comment of the state of JEV vaccination in Indonesia in general and the reach of JEV vaccination in Bali.

**Response**: As we have already described (previously in line 249−250), JEV vaccination has been introduced in Bali since 2018, although it has not been widely implemented in Indonesia. Hence, the national coverage is not known. However, the coverage of JEV vaccination in Bali in 2018 was reported to be 93.58% (Sawitri et al., 2018). We have now added this information to the main text (lines 356−357).

3. LN 226, The sentence “80% of Indonesian children have experienced DENV infection at least once before the age of ten, which likely explains the low prevalence of primary JEV infection in our study”. Do the authors mean primary DEN?

**Response**: We apologize for the confusion. By primary JEV infection we mean that the patients had no detectable or low neutralizing antibodies to JEV or any DENV in their S1 specimen as exemplified by WGY 599 and WGY623. In other words, JEV was the first flavivirus that they encountered. We have now revised the text to indicate this (lines 322−324).

4. Pg 6. The final assessment of JEV positivity by IgM and PRNT would be better expressed in a table to facilitate reading.

**Response**: We thank the reviewer for the suggestion. We have now included Table 1 for diagnostic interpretation of the serology testing results.

**Author responses to other reviewer comments from Editor’ email:**

Methods
-Are the objectives of the study clearly articulated with a clear testable hypothesis stated?
-Is the study design appropriate to address the stated objectives?
-Is the population clearly described and appropriate for the hypothesis being tested?
-Is the sample size sufficient to ensure adequate power to address the hypothesis being tested?
-Were correct statistical analysis used to support conclusions?
-Are there concerns about ethical or regulatory requirements being met?

Reviewer #1: The study had clearly stated objectives and design for an observation in a hospital population.  I believe the study reports on a sufficient number of cases to support the findings but the nature of the study does not requirer statistical analyses.  My only comment on design is an editorial one.  Use of RT-PCR for dengue analysis of S1 is described under "Study site, patient recruitment and sample collection".  This is fine, but use of PCR should appear under Methods in the same way that Elisa, PRNT etc. do.  Either the description of Rt-PCR should be moved there under a heading of Rt-PCR or there should be a note in the methods  under an RT-PCR heading that refers to the description in the Study site section.  I think the former is preferable. I have no concerns about ethical conduct of the study.

Reviewer #2: The objective of the study was articulated well and the study design was appropriate for addressing the objective.

Reviewer #3: (No Response)

**Response**: We thank for the suggestion from Reviewer #1. We have now moved the description of RT-PCR assays used in the study to a new section (“DENV NS1 antigen detection and RT-PCR assays”) under Methods.

--------------------

Results
-Does the analysis presented match the analysis plan?
-Are the results clearly and completely presented?
-Are the figures (Tables, Images) of sufficient quality for clarity?

Reviewer #1: I believe that the results are presented clearly and match the analysis plan.  The category criteria for confirmed and probable JE or dengue are, of necessity, complex and the textual explanation of why some probable JE cases are not dengue is critical and helps one understand the small +/- data presented in the Table.  I would suggest, however that the RT-PCR data for dengue be cited in the results as further evidence in support of those probable cases fitting JE more closely, because RT-PCR is highly sensitive and specific in the febrile period for dengue diagnosis.  It is reported in a sentence in the manuscript that there were no positive RT-PCR for dengue or JE.  That is unsurprising for JE as it is quite rare to have high enough sustained viremia to detect JE infection with PCR, but not so with dengue. The fact that PCR was negative for dengue in the Probably JE cases adds to the argument made on the basis of serologic criteria that the JE probable cases were in fact more likely to be JE.  I think it helps your case if you state that clearly in this section of the results.

Reviewer #2: The results of the study were presented clearly, however, there was room for inclusion of more information.

Reviewer #3: (No Response)

**Response**: We thank Reviewer #1 for the suggestion. We have now added a statement to indicate that the JE probable cases identified on the basis of serologic criteria were more likely to be JE rather than dengue because of the absence of DENV RNA detectable by highly sensitive RT-PCR in the S1 specimens (lines 249−251). We have also included more information regarding dengue clinical data (Table 3) for comparison with JE cases as suggested by Reviewer #2.

--------------------

Conclusions
-Are the conclusions supported by the data presented?
-Are the limitations of analysis clearly described?
-Do the authors discuss how these data can be helpful to advance our understanding of the topic under study?
-Is public health relevance addressed?

Reviewer #1: The conclusions are supported by the data and I believe, as noted above, reporting of RT-PCR negative dengue tests in the results would make an even stronger case.  There is no real discussion of limitations other than the difficulty of flavivirus serologic cross-reactivity. The small numbers don't allow for any stratification of results and statistical analysis and that should be described as a limitation.  I think the finding of a clinical, non-encephalitic state for JE, along with that described in reference 22, is very important. Most people think JE presents only as severe illness or asymptomatic infection.  It is helpful to understand that there is, not surprisingly, an intermediate circumstance.  My last comment is that the authors should consider that the odd adult distribution is likely due to the fact that this is numerator only study.  Seeming predominance of adult cases of JE is common where only numerator data is available. The 15-20% of individuals who are not infected as children and acquire the virus later in life can be quite numerous in a population. In most circumstances where vaccine has not been broadly applied, adjusting the numerator data by age stratified population denominators shows that the incidence is much higher in children under 15 years of age.  The authors should address the lack of population denominator data as another key limitation of the study.

Reviewer #2: The conclusion of the study was supported by the data presented.

Reviewer #3: (No Response)

**Response**: We thank Reviewer #1 for the comments and suggestions. We have now included under Discussion section the limitation of our study particularly regarding limited number of the identified cases as well as the lack of population denominator (lines 347−353).

**Text added:** This study is limited by the number of non-encephalitic JE cases identified which does not allow for a sound stratified analysis of the results particularly regarding the clinical features. Furthermore, the higher prevalence of adult over children cases identified in the study might be due to lack of appropriate population denominator data. The incidence of non-encephalitic JE might potentially be higher in children if the data from this study were adjusted by age stratified population denominator (i.e. the number of susceptible children during the study period).

--------------------

Editorial and Data Presentation Modifications?
Use this section for editorial suggestions as well as relatively minor modifications of existing data that would enhance clarity. If the only modifications needed are minor and/or editorial, you may wish to recommend “Minor Revision” or “Accept”.

Reviewer #1: I felt that the editorial suggestions that I have are better addressed in the context of the questions posed for review in the above sections and have taken that approach.  I have no more suggestions here.

Reviewer #2: (No Response)

Reviewer #3: (No Response)

**Response**: We thank the reviewers for the comments.
--------------------

Summary and General Comments
Use  this section to provide overall comments, discuss strengths/weaknesses of the study, novelty, significance, general execution and scholarship. You may also include additional comments for the author, including concerns about dual publication, research ethics, or publication ethics. If requesting major revision, please articulate the new experiments that are needed.

Reviewer #1: I think this is of high significance and am grateful to see that the study has been undertaken.  I have seen such information from fever surveillance in one country where clinical symptoms were not readily available.  This study makes it clear that these cases are very likely JE and that they have little symptomatology other, than the 30% of cases with headache, that would make any one think of JE encephalitis. It is very helpful to broaden the understanding of the spectrum of JE disease.

Reviewer #2: This would be a missed opportunity if some additional results were not included as mentioned in the review comments.

Reviewer #3: The draft manuscript describes a retrospective analyses of paired-serum  samples collected from febrile, not encephalitic, patients in Bali, Indonesia. A significant proportion of these samples demonstrate seroconversion to JEV, perhaps underscoring the need of more robust surveillance in the region and the need for JEV vaccinations. Nonetheless, as is, the draft lacks more thorough and consistent testing to properly provide for evidence of JEV seroconversions. Before acceptance, authors should be given an opportunity to address findings in attached letter.

**Response**: We thank the reviewers for the comments and suggestions. As the Reviewer #1 and #2 have suggested, we have now included the clinical data of all confirmed dengue cases identified from the cohort to provide comparative description with confirmed and probable JE cases clinical data (Table 3 and lines 278−284, 328−336). Regarding Reviewer #3 concern, we have now provided more descriptions and discussions regarding the diagnosis of confirmed and probable JE on the basis of serological findings. These includes Fig 1 to provide contextual explanation of the systematic testing flow, Table 1 to provide clear diagnostic interpretation, and text in lines 243−251 to provide further arguments of JE diagnosis as opposed to dengue.

**Reference**:

Bradt, V., Malafa, S., von Braun, A., Jarmer, J., Tsouchnikas, G., Medits, I., Wanke, K., Karrer, U., Stiasny, K., and Heinz, F.X. (2019). Pre-existing yellow fever immunity impairs and modulates the antibody response to tick-borne encephalitis vaccination. Npj Vaccines *4*, 38.

Chan, K.R., Wang, X., Saron, W.A.A., Gan, E.S., Tan, H.C., Mok, D.Z.L., Zhang, S.L.X., Lee, Y.H., Liang, C., Wijaya, L., et al. (2016). Cross-reactive antibodies enhance live attenuated virus infection for increased immunogenicity. Nat. Microbiol. *1*, 1–10.

Chanama, S., Sukprasert, W., Sa-ngasang, A., A-nuegoonpipat, A., Sangkitporn, S., Kurane, I., and Anantapreecha, S. (2005). Detection of Japanese Encephalitis (JE) virus-specific IgM in cerebrospinal fluid and serum samples from JE patients. Jpn. J. Infect. Dis. *58*, 294–296.

Lanciotti, R.S., Kosoy, O.L., Laven, J.J., Velez, J.O., Lambert, A.J., Johnson, A.J., Stanfield, S.M., and Duffy, M.R. (2008). Genetic and serologic properties of Zika virus associated with an epidemic, Yap State, Micronesia, 2007. Emerg. Infect. Dis. *14*, 1232–1239.

Makino, Y., Tadano, M., Saito, M., Maneekarn, N., Sittisombut, N., Sirisanthana, V., Poneprasert, B., and Fukunaga, T. (1994). Studies on Serological Cross-Reaction in Sequential Flavivirus Infections. Microbiol. Immunol. *38*, 951–955.

Megawati, D., Masyeni, S., Yohan, B., Lestarini, A., Hayati, R.F., Meutiawati, F., Suryana, K., Widarsa, T., Budiyasa, D.G., Budiyasa, N., et al. (2017). Dengue in Bali: Clinical characteristics and genetic diversity of circulating dengue viruses. PLoS Negl. Trop. Dis. *11*, 1–15.

Montoya, M., Collins, M., Dejnirattisai, W., Katzelnick, L.C., Puerta-Guardo, H., Jadi, R., Schildhauer, S., Supasa, P., Vasanawathana, S., Malasit, P., et al. (2018). Longitudinal analysis of antibody cross-neutralization following zika virus and dengue virus infection in Asia and the Americas. J. Infect. Dis. *218*, 536–545.

Robinson, J.S., Featherstone, D., Vasanthapuram, R., Biggerstaff, B.J., Desai, A., Ramamurty, N., Chowdhury, A.H., Sandhu, H.S., Cavallaro, K.F., and Johnson, B.W. (2010). Evaluation of three commercially available Japanese encephalitis virus IgM enzyme-linked immunosorbent assays. Am. J. Trop. Med. Hyg. *83*, 1146–1155.

Sawitri, A.A.S., Yuliyatni, C.D., Ariawan, I.M.D., and Sari, K.A.K. (2018). Coverage evaluation of Japanese encephalitis supplementary immunization activities (JE SIA) in Bali island 2018. 1–89.

Solomon, T., Dung, N.M., Kneen, R., Gainsborough, M., Vaughn, D., and Khanh, V.T. (2000). Japanese encephalitis. J. Neurol. Neurosurg. Psychiatry *68*, 25–30.

Wong, R., and Bhattacharya, D. (2019). Basics of memory B-cell responses: lessons from and for the real world. Immunology *156*, 120–129.

World Health Organization (2006). Japanese encephalitis surveillance standards. 45–51.
